# Cryptosporidiosis Associated with Consumption of Unpasteurized Goat Milk — Idaho, 2014

**Published:** 2015-02-27

**Authors:** Mariana Rosenthal, Randi Pedersen, Scott Leibsle, Vincent Hill, Kris Carter, Dawn M. Roellig

**Affiliations:** 1 Epidemic Intelligence Service, CDC; 2 Bureau of Communicable Disease Prevention, Idaho Department of Health and Welfare; 3 Southwest District Health (Idaho); 4 Idaho State Department of Agriculture; 5 Division of Foodborne, Waterborne, and Environmental Diseases, National Center for Emerging and Zoonotic Infectious Diseases, CDC

On August 27, 2014, the Idaho Department of Health and Welfare’s Division of Public Health (DPH) was notified of two cases of cryptosporidiosis in siblings aged <3 years. Idaho’s Southwest District Health (SWDH) investigated and found that both children had consumed raw (unpasteurized) goat milk produced at a dairy licensed by the Idaho State Department of Agriculture (ISDA) and purchased at a retail store. Milk produced before August 18, the date of illness onset, was unavailable for testing from retail stores, the household, or the dairy. Samples of raw goat milk produced on August 18, 21, 25, and 28, taken from one opened container from the siblings’ household, one unopened container from the retailer, and two unopened containers from the dairy, all tested positive for *Cryptosporidium* by real-time polymerase chain reaction (PCR) at a commercial laboratory. On August 30, ISDA placed a hold order on all raw milk sales from the producer. ISDA and SWDH issued press releases advising persons not to consume the raw milk; SWDH issued a medical alert, and Idaho’s Central District Health Department issued an advisory to health care providers about the outbreak.

All seven of Idaho’s Public Health Districts and DPH continued to monitor cryptosporidiosis reports submitted from Idaho health care providers and laboratories statewide as required by Idaho law. Public Health Districts investigated reports by interviewing ill persons or their parents using a standardized questionnaire. After the hold order, SWDH and the Central District Health Department identified nine ill persons in four households. Four persons who had regularly consumed raw goat milk produced before August 18 experienced symptoms of gastroenteritis, and five household members who had not consumed the milk experienced onsets of symptoms of gastroenteritis 3–8 days after the first household member became ill. No other common exposures were identified. CDC case definitions for cryptosporidiosis were used (1). In total, the 11 ill persons were aged 2 months–76 years (median = 11 years); six were female. One patient was hospitalized. Stool specimens were obtained in three primary cases (i.e., illnesses in those who drank the raw goat milk) and three secondary cases (i.e., illness in contacts of those who drank the raw goat milk); CDC isolated *Cryptosporidium parvum* subtype IIaA16G3R1 from all six. The last reported outbreak-associated illness was a secondary case with an onset date of September 3.

In addition to the four tested milk samples from containers, five of five milk samples collected along the production line on September 2 tested positive for *Cryptosporidium* by PCR at the commercial laboratory. Testing of all nine milk samples (four from containers and five from the production line) at CDC for *Cryptosporidium* by PCR and direct fluorescent antibody test was negative. CDC and the commercial laboratory collaborated to validate the negative result by using sequencing to determine that false-positive results at the commercial laboratory were likely caused by goat DNA amplification during PCR. An inspection of the dairy did not reveal any obvious contamination sources. Water from the producer’s well tested negative at Idaho Bureau of Laboratories for *Cryptosporidium* by direct fluorescent antibody test after ultrafiltration. Goat stool was unavailable for testing. Negative results led ISDA to release the hold order on September 18.

Epidemiologic evidence implicated contaminated raw goat milk as the outbreak source. It was not possible to obtain confirmatory laboratory evidence of milk contamination. Milk consumed before illness onset was unavailable for testing and could have been subjected to a single, undetected contamination event. No other common source was identified, and isolation of the identical *Cryptosporidium* genotype from ill persons did not disprove a common source. This outbreak highlights an infrequently reported cryptosporidiosis risk from unpasteurized milk (2,3), the value of sequencing to validate PCR protocols, the utility of genotyping *Cryptosporidium* isolates for strengthening epidemiologic evidence, and the risk for secondary transmission of *Cryptosporidium*. An increasing number of enteric outbreaks are associated with raw milk consumption (4,5). Resources for consumers, health care providers, and public health officials regarding risks from raw milk consumption are available at http://www.cdc.gov/foodsafety/rawmilk/raw-milk-index.html.
